# Exceptional high fatigue strength in Cu-15at.%Al alloy with moderate grain size

**DOI:** 10.1038/srep27433

**Published:** 2016-06-06

**Authors:** Rui Liu, Yanzhong Tian, Zhenjun Zhang, Xianghai An, Peng Zhang, Zhefeng Zhang

**Affiliations:** 1 Shenyang National Laboratory for Materials Science, Institute of Metal Research, Chinese Academy of Sciences, 72 Wenhua Road, Shenyang 110016, P.R. China; 2 School of Aerospace, Mechanical and Mechatronic Engineering, The University of Sydney, Sydney, New South Wales 2006, Australia

## Abstract

It is commonly proposed that the fatigue strength can be enhanced by increasing the tensile strength, but this conclusion needs to be reconsidered according to our study. Here a recrystallized α-Cu-15at.%Al alloy with moderate grain size of 0.62 μm was fabricated by cold rolling and annealing, and this alloy achieved exceptional high fatigue strength of 280 MPa at 10^7^ cycles. This value is much higher than the fatigue strength of 200 MPa for the nano-crystalline counterpart (0.04 μm in grain size) despite its higher tensile strength. The remarkable improvement of fatigue strength should be mainly attributed to the microstructure optimization, which helps achieve the reduction of initial damage and the dispersion of accumulated damage. A new strategy of “damage reduction” was then proposed for fatigue strength improvement, to supplement the former strengthening principle. The methods and strategies summarized in this work offer a general pathway for further improvement of fatigue strength, in order to ensure the long-term safety of structural materials.

The improvement of fatigue strength is crucial for the industrial application of structural materials for ever. In the past several decades, solutions of this problem are tightly connected to the enhancement of tensile properties, as it is generally accepted that the improvement of tensile strength usually leads to a corresponding improvement of fatigue strength^1^,^2^,^3^. Accordingly, a variety of strengthening methods, including alloying^4^, grain refinement^5^ and heat treatment^6^ were carried out for higher fatigue strength. However, this relationship seems not remain for long: at high-strength level or in the very high cycle fatigue (VHCF)^7^,^8^ regime, the fatigue strength tends to keep constant^9^ or even decreases^10^,^11^ by further increasing the tensile strength. The transitions of crack initiation mechanisms and dominant fatigue damage mechanisms^12^ are considered as the underlying factors that changed the initial linear relation. Based on these studies, a parabolic relation between fatigue strength and tensile strength was proposed and confirmed by large amounts of statistical data^13^.

Above non-monotonic relation between tensile strength and fatigue strength indicate the existence of other factors influencing the value of fatigue strength, and it is not applicable to build direct relation between tensile properties and fatigue properties. In comparison with the tensile properties which can be considered as a reflection of materials overall behaviors, the fatigue properties are usually influenced by local weaknesses, such as micro-cracks and inclusions^14^, so these initial damages should be avoided as possible. What’s more, microstructure instability^15^,^16^,^17^, which can cause local softening during cyclic loading, should also be taken into consideration. Concerning the issues above, the fatigue strength is a comprehensive reflection of not only the macroscopic tensile behaviors but also the microscopic damage conditions. In this case, it becomes difficult to predict the fatigue strength, yet brings new chances to propose new theories in the same time.

For years, systematic studies on fatigue behaviors of face-center-cubic alloys (especially α-Cu-Al alloys) have been conducted in our research group^4^,^18^,^19^,^20^,^21^. According to previous works, two methods can effectively im’prove the fatigue properties: alloying^4^,^20^,^21^ and grain refining^18^,^19^. For the first method, the alloying elements can contribute more than the solution strengthening effect. By adding the components which help decrease the stacking fault energy (SFE) of alloys (as Al for Cu-Al alloys), dislocation cross-slip can be suppressed, leading to the improvement of both the microstructure stability and the deformation reversibility^4^,^18^,^20^, thus better fatigue damage resistances can be obtained. For the second method, severe plastic deformation (SPD) was conducted in order to refine the grains down to the nano-scale^22^,^23^. The fatigue strength was indeed improved, however, in comparison with the significant increase of the tensile strength, the range of fatigue strength improvement is rather limited^19^. It is probably due to the original damage (including high-density defects^24^ and micro-cracks^25^) induced by SPD methods^26^. Further studies showed that the annealing process following SPD methods can help diminishing the original damage through recrystallization^27^,^28^; by cold rolling and annealing process^29^, better combination of tensile strength and ductility can be achieved. Therefore, the microstructure optimization is carried out here for achieving higher fatigue strength.

In this study, an α-Cu-Al alloy with high Al content (Cu-15at.%Al, which displayed the best fatigue properties in Ans research^18^,^19^) is chosen, with the cold-rolling and annealing process^29^ (which brings smaller deformation damage in comparison to SPD methods), aiming at improving fatigue strength a step further.

## Results

### Improvement of fatigue strength

**Figure 1:**
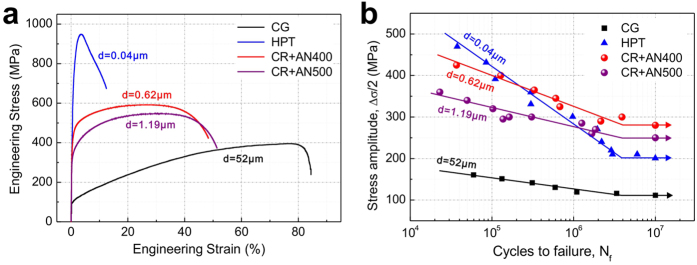
Tensile curves and S-N curves of Cu-15at.%Al. (**a**) Engineering stress-strain curves. (**b**) Relationships between stress amplitude and fatigue life. CG: coarse-grained specimens, HPT: specimens fabricated by high-pressure torsion, CR+AN400/500: specimens fabricated by cold rolling and annealing at 400/500 ^o^C; results of CG and HPT were from the work of An *et al.*^18^.

Cu-15at.%Al alloys with ultrafine grains (UFGs) produced by cold rolling and subsequent annealing processes 29 have been investigated in this study. To obtain fully recrystallized microstructures with grain sizes as small as possible, an annealing process (400 °C for 5 min) was chose after a series of attempts, which could produce equiaxed grains with an average size of 0.62 μm. Another annealing process (500 °C for 10min) corresponding to an average grain size of 1.19 μm has also been conducted as a control group. In addition, coarse grains (CGs, 52 μm, by high-temperature annealing) and nano-grains (NGs, 0.04 μm, by HPT) reported by An *et al.*^18^ were also taken into consideration. Figure 1a shows the tensile stress-strain curves of these four kinds of specimens. A trade-off relation between tensile strength and uniform elongation can be readily noticed: by decreasing the average grain size, the tensile strength monotonically increases while the uniform elongation continuously decreases. In comparison with the CGs and NGs, the UFG specimens displayed rather high yield strength with promising uniform elongation. The S-N curves and fatigue strengths are summarized in Fig. 1b. It is obvious that both the slope of the S-N curves and the fatigue strength of the present two recrystallized materials are remarkably improved compared to that of the HPT one. As a result, the material with the highest fatigue strength among the four kinds of specimens is the UFG alloy with a grain size of 0.62 μm (280 MPa), which has neither the highest tensile strength (NGs, 200 MPa) nor the best plasticity (CGs, 110 MPa) 18. Moreover, even the specimens with a grain size of 1.19 μm attained a rather high fatigue strength of 250 MPa. Compared to the former results, further improvement of fatigue strength has been successfully achieved in the two UFG alloys in this study.

### Reasons for the improvement: related macroscopic properties

**Figure 2:**
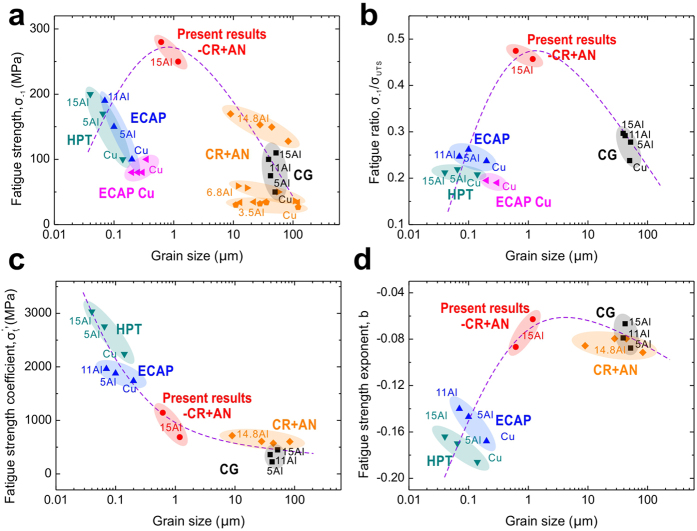
Relationships between fatigue properties and grain size. (**a**) Fatigue strength and grain size. (**b**) Fatigue ratio and grain size. (**c**) Fatigue strength coefficient *σ*_*f*_′ and grain size. (**d**) Fatigue strength exponent *b* and grain size. *σ*_*f*_′ and *b* are parameters in the Basquin equation. CG/ECAP/HPT: data from the work of An *et al.*^18^, the coarse grain/ECAP/HPT structures; ECAP Cu: data from the work of Agnew^30^, Vinogradov^31^, Hoppel^32^ and Han^33^, the UFG copper produced by ECAP; CR + AN: data from the work of Nishimura *et al.*^34^, materials fabricated by cold rolling and annealing. Present results-CR+AN: data in this study, specimens fabricated by cold rolling and annealing.

Figure 1b indicates that the S-N curves fitted well with the Basquin equation: Δ*σ*/2 = *σ*_*f*_′*·*(2*N*_*f*_)^*b*^. A group of relations between mechanical parameters and grain size are shown in Fig. 2 and the corresponding data are listed in Table 1. To make the comparison more objective and impartial, research results of Agnew^30^, Vinogradov^31^, Hoppel^32^, Han^33^ and Nishimura^34^ have also been taken into consideration. As displayed in this figure, except for the inverse relationship of the fatigue strength coefficient in Fig. 2c (σ_*f*_′, the intercept of S-N curve according to the Basquin equation), the other three parameters, including the fatigue strength (*σ*_−1_, Fig. 2a), the fatigue ratio (*σ*_−1_/*σ*_UTS_, Fig. 2b) and the fatigue strength exponent (*b*, the slop of S-N curve according to the Basquin equation, Fig. 2d), show hump-shaped trend and all the peak values are from our present results. According to these conditions, several conclusions could be drawn as follows. **i)** This further improvement of fatigue strength should not be mainly owing to strengthening, as the fatigue strength coefficient *σ*_*f*_′, which is known as a parameter directly related to the tensile strength^35^, showed a completely different trend with the fatigue strength (Fig. 2a,c). **ii**) The value of fatigue ratio *σ*_-1_/*σ*_UTS_, a reflection of the comparison between tensile and fatigue properties, remarkably influences the tendency of fatigue strength (Fig. 2a,b). The peak value broke up the common positive correlation between the tensile strength and the fatigue strength. **iii**) The change of the fatigue ratio is possibly correlated with the microscopic mechanisms of fatigue damage, which could be partially reflected by the fatigue strength exponent *b*^36^. The similar trends of the fatigue ratio and fatigue strength exponent *b* reflected this possibility (Fig. 2b,d). In order to confirm this speculation, a series of microscopic observations were carried out; corresponding results will be introduced in the following section.

### Reasons for the improvement: latent microscopic mechanisms

For ordinary SPD materials, the fatigue strength exponent *b* is usually much smaller than the undeformed state^5^, thus causing the shrink of fatigue ratio *σ*_-1_/*σ*_UTS_. According to the former investigations^18^, fatigue strength exponent *b* is a reflection of fatigue damage mechanism; the microstructure instability and initial damage of SPD materials can be considered as the two main reasons for its decrease. In this section, the original microstructures as well as microstructures after fatigue test were carefully observed by EBSD and TEM, to evaluate the cyclic stability and initial damage level of our materials.

**Figure 3:**
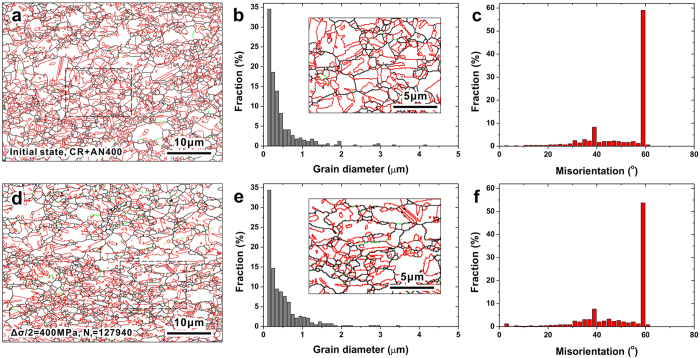
EBSD results of the Cu-15at.%Al alloy with a mean grain size of 0.62 μm before and after fatigue tests. (**a–c**) EBSD results of the initial state, CR+AN400: cold rolling and annealing at 400 °C. (**d–f**) EBSD results after fatigue fracture, stress amplitude and fatigue life were noted in figure (**d**). (**a,d**) Images of boundary distributions. The black, green and red lines represent high-angle grain boundaries, low-angle grain boundaries and twin boundaries, respectively. Corresponding images with a larger magnification are shown in (**b,e**). (**b,e**) Distributions of grain diameters. (**c,f**) Distributions of boundary misorientation angles.

The EBSD results in Fig. 3 give a clear description of the grain morphology and boundary conditions of the UFG material with a mean grain size of 0.62 μm, which attains the highest fatigue strength. Brief comparisons between conditions before and after cyclic loading can be made between Fig. 3a–f. According to the results of the original state (Fig. 3a–c), the grains are equiaxed and substantially uniform. Low-angle grain boundaries (green lines in Fig. 3a with the misorientation <15° in Fig. 3c) can rarely be observed, which means that the material has been fully recrystallized with less initial defects retained. In contrast, the proportion of twin boundaries is rather large (~60%, Fig. 3c); these low energy interfaces with excellent thermostability are beneficial to the structure stability under cyclic loading^37^. Figure 3d–f show corresponding results after fatigue tests at a relatively large stress amplitude (400 MPa). Comparing with the initial state, the grain morphology rarely changed (Fig. 3a,d), as well as the distribution of grain sizes (Fig. 3b,e). Local grain coarsening wasn’t observed, which often occurs in the SPD materials^5^,^18^. For the misorientation conditions (Fig. 3c,f), the proportion of boundaries with small misorientation angles (<5°) increased and the twin boundaries decreased slightly. Besides the effect of different regions for observation, the formation of low-angle boundaries by dislocation accumulation, together with dislocation-twin interaction during cyclic loading, are possible explanations for above changes. Despite these delicate differences, on the whole, the grain morphology and boundary conditions rarely change during cyclic loading.

**Figure 4:**
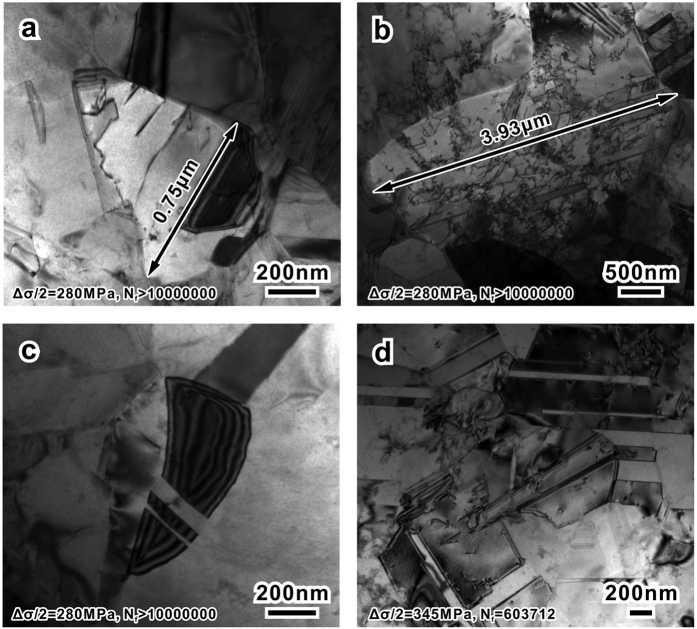
TEM images of the alloy with a mean grain size of 0.62 μm after fatigue tests. (**a,b**) Different dislocation densities of relative (**a**) small and (**b**) large grains. (**c,d**) The stable state of annealing twin boundaries, in both (**c**) small and (**d**) large grains. Stress amplitude and corresponding fatigue life were noted in each figure.

TEM characterizations displayed in Fig. 4 further confirmed above analysis. In general, the grains are rather clean, no micro-cracks or inclusions were observed. Even few dislocations could be found in grains with smaller size (Fig. 4a) after the fatigue tests. In relatively larger grains, more dislocations initiated and dispersedly distributed as typical planar-slip patterns (Fig. 4b). This distinction among grains with different sizes indicates that the fatigue damage prefers to occur in larger grains; as the range of grain size is rather narrow for our materials (Fig. 3b,e), this kind of damage concentration is not so obvious. As for the conditions of grain boundaries, coherent annealing twin boundaries with few defects can be frequently observed in grains with various sizes (Fig. 4c,d). In contrast with typical deformation twins containing many defects and steps^38^ that would lead to de-twinning^39^, or the imperfect nano-scaled twins which could cause the twin-assisted grain growth^40^, these micron-sized annealing twin boundaries possess perfect coherent structure with lower energy state and better thermal stability^41^,^42^,^43^. According to the microstructure observation, this recrystallized microstructure contain fewer initial damages and display better cyclic stability in comparison with typical SPD structures^15^,^16^.

## Discussion

### Microstructure optimization: an effective method of fatigue strength improvement

According to above results, the recrystallized UFG microstructures produced by cold rolling and annealing gained larger fatigue strength than both the NGs attained by SPD processes and the CGs obtained by high temperature annealing. The superiorities of this UFG structure could be summarized as follows based on former tests and observations.

Comparing with the CG structures produced by high temperature annealing, this UFG structure contains grains with much smaller size. The refinement of grains increased the strength and the overall damage resistance, which are reflected in the notable increase of the fatigue strength coefficient *σ*_*f*_′ (Fig. 2c). Otherwise, as both the UFGs and the CGs are fully recrystallized grains, their initial damage degrees and cyclic damage mechanisms should be similar, as reflected in the similar values of the fatigue strength exponent *b* (Fig. 2d). Under this premise, the CGs, which contains more grains in large sizes are easier to deform plastically in these large grains, while the relatively narrow grain size distributions of the UFGs can help prevent the local strain concentration. In this way, the UFGs gain a remarkable improvement of the fatigue strength in comparison with the CGs.

In comparison with the NG structures formed by SPD methods, the larger grain size of the UFG structures can not contribute to the final improvement of the fatigue strength through strengthening method, just as the decreasing fatigue strength coefficient *σ*_*f*_′ (Fig. 2c) shows. However, the UFG structures possess lower initial damage degree and higher microstructure stability, thus leading to a much larger fatigue strength exponent *b* (Fig. 2d). Firstly, the weakened initial damage is attributed by the relative mild deformation manner (cold rolling) and the subsequent annealing process, which decreased the density of dislocations and vacancies through recrystallization. According to the former studies, the existence of local damages produced by SPD methods such as micro-cracks, shear bands and oversaturated defects would cause the local stress concentration^44^,^45^, which could accelerate the initiation and propagation of fatigue cracks^46^. In contrast, the recrystallized UFG structures attain better resistance to fatigue damage due to the clean grains with few initial defects. Secondly, the enhanced microstructure stability should be mainly owing to the reducing of unstable defects with high energy states^47^ through annealing process^29^,^48^. The localized grain coarsening, which could happen in SPD structures during cyclic loading^15^, can be effectively prevented in our UFG structures. In this way, the recrystallized UFG structures gained better resistance to fatigue damage localization. In general, the increase of the fatigue strength exponent *b* displayed a much stronger influence than the decreased fatigue strength coefficient *σ*_*f*_′, resulting in an optimized fatigue properties of the UFG alloys.

**Figure 5:**
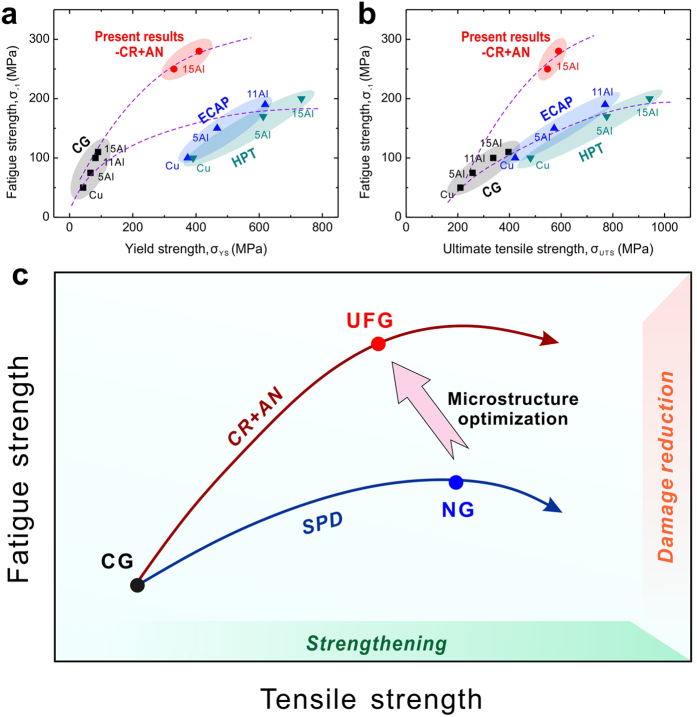
Relationships between fatigue properties and tensile properties: Strategies for the fatigue strength improvement. (**a**) Relationships between fatigue strength and yield strength. (**b**) Relationships between fatigue strength and ultimate tensile strength. Different paths varied with grain refinement processes were shown as curves in figures. CG/ECAP/HPT: data from An *et al.*^18^, the coarse grain/ECAP/HPT specimens; present results-CR+AN: data in this study, specimens fabricated by cold rolling and annealing. (**c**) Summarization of the effective method (microstructure optimizing) and strategies (strengthening and damage reduction) for the improvement of fatigue strength. CG: coarse-grain; UFG: ultrafine grain; NG: nano-grain; CR+AN: cold rolling and annealing; SPD: severe plastic deformation.

According to above analysis, the optimization of microstructures can effectively improve the fatigue strength. Different from alloying and grain refining, the microstructure optimizing can create new path for the fatigue strength improvement. As displayed in Fig. 5a,b, from the original CG state to different conditions of the refined grains, the values of fatigue strength fall on two different curves corresponding to the two refining processes: i) SPD and ii) cold rolling plus subsequent annealing. Obviously, the latter is more effective on the fatigue strength improvement than the former. While the single grain refining method usually put emphasis on strengthening effect, the microstructure optimization concerns mainly about the efficiency of fatigue strength improvement, which can be evaluated by the fatigue ratio. As the relationships shown in Fig. 5c, through optimization, the fatigue ratio can be increased, with an upward rotation of the former grain refining curve.

### General strategies for fatigue strength improvement

For years, strengthening is the widely accepted strategyfor fatigue strength improvement^1^,^2^,^3^, even though it has been proved to be imperfect^9^,^10^,^11^. According to the present study, it is obvious that there exists another way besides strengthening, that is reducing the initial damage and dispersing the accumulated damage, which can be summarized as damage reduction (Fig. 5c). Both strategies have unignorable influence on the value of fatigue strength. In other words, either of their effects on fatigue strength improvement is limited when they work separately; proper cooperation of the two strategies can function effectively.

It is worth noting that both strategies function in a variety of forms and scales, widely from atomic-scale up to meso-scale. For strengthening, defects in different dimensions and scales can help improve the tensile strength to different levels. Solute atoms, dislocations and boundaries, although in varied forms and through various mechanisms, share the same function of strengthening. For damage reduction, it can be roughly classified into damage decrease and damage dispersion: the former puts emphasis on the absolute quantity of damage, and the latter focuses on the distribution of damage. Fatigue damage can be diminished by eliminating the initial damage (changing the density of original defects) or improving the deformation reversibility (changing the deformation mechanisms via alloying^20^). Damage dispersion is realized mainly by improving the homogeneity of fatigue damage accumulation, which could also be achieved in different forms including the diminish of micro-cracks (avoiding over-severe deformation processes), and the decrease of extra-large grains (increasing the homogeneity in crystalline sizes as well as their cyclic stability). In this way, the two strategies would provide a wealth of possibilities for the future studies on the fatigue strength improvement.

In summary, successful examples, practical methods as well as general principles for the improvement of fatigue strength are proposed in this study. We have designed and fabricated fully recrystallized Cu-15at.%Al alloy with mean grain size of ~0.62 μm by conventional cold rolling and annealing process. Exceptional high fatigue strength of 280 MPa and a high fatigue ratio of 0.475 were realized, which are much higher than the 110 MPa and 0.278 for the CG counterpart (52 μm), or the 200 MPa and 0.212 for the NG counterpart (0.04 μm). This progress should be mainly attributed to the microstructure optimization, which realized the reduction of initial damage and the dispersion of accumulated damage. In this way, the common strategy relying only on the improvement of tensile strength has been modified; damage reduction, as well as strengthening, was proposed as another strategy for fatigue strength improvement. We hope these findings could shed light on the further improvement of fatigue strength of structural materials for long-term operation.

## Methods

### Materials fabrication

The Cu-15at.%Al alloy we used in this work was initially melted and casted with 99.99 wt.% pure Cu and 99.99 wt.% pure Al. The as-cast Cu-Al alloy was deformed into rods with 32.5 mm in diameter by drawing process, and then cold-rolled into 7.4 mm thick plates. Following annealing processes were conducted in salt bath: one group of specimens were annealed at 400 °C for 5 min, the other group were annealed at 500 °C for 10 min. Materials after annealing were quenched in water. Fully recrystallized grains with average sizes of 0.62 μm and 1.19 μm were obtained through above processes (the average grain size was measured by the “line intercept method”, according to the microstructures observed by electron backscattered diffraction, EBSD; all the high-angle grain boundaries including twin boundaries were counted).

### Mechanical properties test

The specimens were cut from the cold-rolled and annealed sheets by a wire cutting machine into a dog-bone shape, which has a gauge dimension of 6 mm × 3 mm × 3 mm, with the tension/compression axis parallel to the rolling direction. Uniaxial tensile tests were carried out on an Instron 5982 tensile instrument at aninitial strain rate of 5 × 10^−4^ s^−1^. The strain was measured by an extensometer throughout the tensile test. Each test was repeated at least 3 times to ensure the repeatability of the results. Stress-controlled push-pull fatigue tests were then carried out on an Instron 8871 testing machine with a stress ratio of −1 and a stable frequency of 30 Hz. Both the tensile and the fatigue tests were conducted at room temperature in air.

### Microstructure characterization

Microstructures of specimens before and after fatigue tests were observed by EBSD and transmission electron microscopy (TEM). EBSD observations were carried out on a LEO Supra 35 field emission scanning electron microscope (SEM), with the main operating parameters listed as follows: the operating voltage is 20 kV, the step size of EBSD is 80 nm, and the clean-ups of the EBSD data were performed to diminish the point of zero solutions. The specimens were prepared by mechanical polishing and electro-polishing in a solution of H_3_PO_4_:C_2_H_5_OH:H_2_O = 1:1:2 (vol.) with a voltage of 8–10 V at room temperature. TEM observations were carried on with an FEI Tecnai F20 microscope at an operating voltage of 200 kV. Thin foils for TEM observations were firstly cut from the fatigue specimens parallel to the axis (the rolling direction) by a wire cutting machine, with an original thickness of 300 μm. Then they were mechanically polished to about 50 μm thick, followed by twin-jet polishing in a solution of H_3_PO_4_:C_2_H_5_OH:H_2_O = 1:1:2 (vol.) with a voltage of 8–10 V at −6 °C.

## Additional Information

**How to cite this article**: Liu, R. *et al.* Exceptional high fatigue strength in Cu-15at.%Al alloy with moderate grain size. *Sci. Rep.* **6**, 27433; doi: 10.1038/srep27433 (2016).

## Figures and Tables

**Table 1:** Fatigue properties of pure Cu and Cu-Al alloys with different grain sizes.

Material conditions		Microstructure conditions	Fatigue properties			
Processing method	Content of Al (at.%)	Grain size (μm)	Fatigue strength (MPa)	Fatigue strength coefficient *σ*_*f*_′ (MPa)	Fatigue strength exponent *b*	Fatigue ratio
CR+AN400	15	0.618	280	1144	−0.087	0.475
CR+AN500	15	1.188	250	687	−0.063	0.457
CG^18^	0	50.00	50	–	–	0.238
	5	42.00	75	225	−0.067	0.292
	11	39.00	100	361	−0.079	0.298
	15	52.00	110	448	−0.088	0.278
ECAP^30^	0	0.250	80	–	–	–
ECAP^31^	0	0.200	80	–	–	0.195
ECAP^32^	0	0.350	100	–	–	–
ECAP^33^	0	0.300	80	–	–	0.190
ECAP^18^	0	0.200	100	1737	−0.168	0.237
	5	0.100	150	1878	−0.147	0.262
	11	0.070	190	1962	−0.140	0.247
HPT^18^	0	0.140	100	2238	−0.186	0.208
	5	0.065	170	2754	−0.170	0.219
	15	0.040	200	3032	−0.164	0.212
CR+AN^34^	0	122.0	27	–	–	–
		36.00	34	–	–	–
		28.00	32	–	–	–
		11.00	30	–	–	–
	3.5	120.0	34	–	–	–
		34.00	35	–	–	–
		22.00	36	–	–	–
		13.00	34	–	–	–
	6.8	108.0	36	–	–	–
		57.00	50	–	–	–
		17.00	56	–	–	–
		12.00	60	–	–	–
	14.8	83.00	128	602	−0.091	–
		44.00	150	573	−0.080	–
		28.00	153	605	−0.079	–
		9.00	170	715	−0.086	–

Abbreviations: CR+AN400/500: cold rolling and annealing at 400/500 °C; CG: coarse grain; ECAP: equal-channel angular pressing; HPT: high-pressure torsion; CR+AN: cold rolling and annealing.
